# Effectiveness of five artemisinin combination regimens with or without primaquine in uncomplicated falciparum malaria: an open-label randomised trial

**DOI:** 10.1016/S1473-3099(10)70187-0

**Published:** 2010-09-09

**Authors:** Frank Smithuis, Moe Kyaw Kyaw, Ohn Phe, Thein Win, Pyay Phyo Aung, Aung Pyay Phyo Oo, Arkar Linn Naing, Mya Yee Nyo, Naing Zaw Htun Myint, Mallika Imwong, Elizabeth Ashley, Sue J Lee, Nicholas J White

**Affiliations:** a Médecins sans Frontières—Holland, Yangon, Myanmar; b Medical Action Myanmar, Yangon, Myanmar; c Faculty of Tropical Medicine, Mahidol University, Bangkok, Thailand; d Centre for Tropical Medicine, Nuffield Department of Clinical Medicine, Churchill Hospital, Oxford, UK

## Abstract

**Background:**

Artemisinin-combination therapy (ACT) is recommended as first-line treatment of falciparum malaria throughout the world, and fixed-dose combinations are preferred by WHO; whether a single gametocytocidal dose of primaquine should be added is unknown. We aimed to compare effectiveness of four fixed-dose ACTs and a loose tablet combination of artesunate and mefloquine, and assess the addition of a single gametocytocidal dose of primaquine.

**Methods:**

In an open-label randomised trial in clinics in Rakhine state, Kachin state, and Shan state in Myanmar (Burma) between Dec 30, 2008, and March 20, 2009, we compared the effectiveness of all four WHO-recommended fixed-dose ACTs (artesunate–mefloquine, artesunate–amodiaquine, dihydroartemisinin–piperaquine, artemether–lumefantrine) and loose artesunate–mefloquine in Burmese adults and children. Eligible patients were those who presented to the clinics with acute uncomplicated *Plasmodium falciparum* malaria or mixed infection, who were older than 6 months, and who weighed more than 5 kg. Treatments were randomised in equal numbers within blocks of 50 and allocation was in sealed envelopes. All patients were also randomly assigned to receive either a single dose of primaquine 0·75 mg base/kg or not. Patients were followed up for 63 days. Treatment groups were compared by analysis of variance and multiple logistic regression. The primary outcome was the 63 day recrudescence rate. This study is registered with clinicaltrials.gov, number NCT00902811.

**Findings:**

155 patients received artesunate–amodiaquine, 162 artemether–lumefantrine, 169 artesunate–mefloquine, 161 loose artesunate–mefloquine, and 161 dihydroartemisinin–piperaquine. By day 63 of follow-up, 14 patients (9·4%; 95% CI 5·7–15·3%) on artesunate–amodiaquine had recrudescent *P falciparum* infections, a rate significantly higher than for artemether–lumefantrine (two patients; 1·4%; 0·3–5·3; p=0·0013), fixed-dose artesunate–mefloquine (0 patients; 0–2·3; p<0·0001), loose artesunate–mefloquine (two patients; 1·3%; 0·3–5·3; p=0·0018), and dihydroartemisinin–piperaquine (two patients 1·3%; 0·3–5·2%; p=0·0012). Hazard ratios for re-infection (95% CI) after artesunate–amodiaquine were 3·2 (1·3–8·0) compared with the two artesunate–mefloquine groups (p=0·01), 2·6 (1·0–6–0) compared with artemether–lumefantrine (p=0·04), and 2·3 (0·9–6·0) compared with dihydroartemisinin–piperaquine (p=0·08). Mixed falciparum and vivax infections were common: 129 (16%) had a mixed infection at presentation and 330 (41%) patients had one or more episodes of *Plasmodium vivax* infection during follow-up. The addition of a single dose of primaquine (0·75 mg/kg) reduced *P falciparum* gametocyte carriage substantially: rate ratio 11·9 (95% CI 7·4–20·5). All regimens were well tolerated. Adverse events were reported by 599 patients, most commonly vomiting and dizziness. Other side-effects were less common and were not related to a specific treatment.

**Interpretation:**

Artesunate–amodiaquine should not be used in Myanmar, because the other ACTs are substantially more effective. Artesunate–mefloquine provided the greatest post-treatment suppression of malaria. Adding a single dose of primaquine would substantially reduce transmission potential. Vivax malaria, not recurrent falciparum malaria, is the main complication after treatment of *P falciparum* infections in this region.

**Funding:**

Médecins sans Frontières (Holland) and the Wellcome Trust Mahidol University Oxford Tropical Medicine Research Programme.

## Introduction

Artemisinin-based combination therapy (ACT) is recommended by WHO for the treatment of uncomplicated falciparum malaria.^1^ The success of this recent policy change will depend on the efficacy of the combination components, high population coverage, low costs, correct dosing, and ensuring good adherence to prescribed treatment. To improve adherence and acceptability, and prevent one drug being taken without its partner, ACTs are preferably formulated in fixed-dose combinations.^2^, ^3^, ^4^, ^5^ Four fixed-dose ACTs are now available: two new combinations (artesunate–mefloquine and artesunate–amodiaquine) now join artemether–lumefantrine and dihydroartemisinin–piperaquine.

Artemisinin and its derivatives reduce gametocyte carriage,^6^ but they do not prevent transmission from gametocytaemia present at the time of treatment.^7^ A single gametocytocidal dose of primaquine was widely recommended in low transmission areas before the introduction of ACTs.^8^ With the greater effects of artemisinins on gametocyte carriage there has been uncertainty whether primaquine should be added to ACT regimens.^9^ This question is of increasing importance as countries move from malaria control to elimination, which will require effective, well tolerated medicines and reduction of transmission.

Our aim was to compare the efficacy of the four available fixed-dose ACTs and the currently used loose tablet combination of artesunate with mefloquine and to assess the effectiveness of adding a single gametocytocidal dose of primaquine.

## Methods

### Patients

Between Dec 30, 2008, and March 20, 2009, we recruited patients into our open-label randomised study at three clinics in Rakhine state in western Myanmar (Burma),^2^, ^3^, ^4^, ^10^ two clinics in Kachin state in northern Myanmar, and one clinic in Shan state in northeast Myanmar. Patients older than 6 months who weighed more than 5 kg and presented with acute uncomplicated *Plasmodium falciparum* malaria (parasite density 500–200 000 parasites per μL) or mixed infection were enrolled into the study after fully informed consent was obtained from them or their carer. Patients were excluded if they were pregnant, had severe malaria, had severe acute malnutrition (weight-for-height below 70% of median with or without symmetrical peripheral oedema), had taken antimalarial drugs within the past 48 h, had taken mefloquine during the past 9 weeks, or had known history of hypersensitivity to any of the study drugs.

### Randomisation

After patients were screened and enrolled into the study, they were stratified prospectively into three age groups (1–4 years, 5–14 years, and older than 14 years). Patients were randomly assigned in equal numbers to receive one of the five different treatments. They were then randomly assigned either a single dose of primaquine 0·75 mg base/kg (Government Pharmaceutical Organisation, Bangkok, Thailand) or not. Treatment allocations were put in sealed envelopes in blocks of 50 for each age-group, and random assignment was achieved by patients drawing an envelope from a box after enrolment. When the box was empty, another 50 envelopes were added.

### Procedures

For the four fixed-dose combinations the standard dosage instructions of the manufacturer for weight and age ranges were followed. For the loose tablets regimen of artesunate plus mefloquine, the target dose was artesunate 4 mg/kg per day for 3 days (total 12 mg/kg) plus mefloquine 25 mg base/kg on day 0; the number of pills given was rounded off to the nearest quarter of a tablet. For fixed dose artesunate–mefloquine hydrochloride we used 25 mg plus 55 mg or 100 mg plus 220 mg tablets, the target dose was 4·0 mg/kg per day plus 8·8 mg/kg per day for 3 days. For fixed-dose artemether–lumefantrine we used 20 mg plus 120 mg tablets twice daily for 3 days, the target dose was 3·3 mg/kg per day plus 19·8 mg/kg per day; patients were advised to consume some fatty food (or mothers were encouraged to breastfeed treated infants) before each dose. For fixed-dose dihydroartemisinin–piperaquine we used 40 mg plus 320 mg or 20 mg plus 160 mg tablets, the target dose was 2·5 mg/kg per day plus 20 mg/kg per day. For fixed dose artesunate–amodiaquine we used 25 mg plus 67·5 mg, 50 mg plus 135 mg, or 100 mg plus 270 mg tablets, the target dose was 4 mg/kg per day plus 10·8 mg base/kg per day.

The first dose was taken under supervision. For children, tablets were crushed and syrup was added. All subsequent doses were self administered. Patients received sealed plastic bags containing the remaining doses and were instructed clearly about their subsequent treatment, emphasising the importance of taking primaquine after food, and taking their medicines even when their symptoms had subsided. Patients were asked to return weekly for 9 weeks for assessment, and at any other time if they became ill. Microscopists examining blood films were unaware of treatment allocation. Haemoglobin was measured on day 63.

Patients with recurrent falciparum malaria were treated with artesunate–mefloquine (loose tablets). Patients who had already received this treatment were given dihydroartemisinin–piperaquine. PCR genotyping was used to distinguish recrudescence from re-infection, by assessment of variable blocks within merozoite surface proteins 1 and 2, and glutamate-rich protein.^11^

Patients who developed intercurrent *Plasmodium vivax* or *Plasmodium malariae* infections received chloroquine (25 mg base/kg) and continued follow-up. If the patients were again positive for *P vivax* 14 days or later, they were retreated with chloroquine and defined as having a new *P vivax* episode (as a result of treatment failure, relapse, or, less likely, a new infection).

### Statistical analysis

A sample size of 160 patients (80 with primaquine) per study group (total 800 patients) allowed a cure rate (100–recrudescence rate) of 95% per group to be estimated with 5% precision, and allowed estimation of effectiveness equivalence with a maximum allowable difference of 10% (90% power and 95% confidence) between groups with a follow-up drop-out rate of up to 20%.

Treatment groups were compared by two-way factor analysis: ANOVA for continuous variables and multiple logistic regression for categorical data. A test for trend was used for comparisons across age groups. Time-to-event outcomes, including time to first recurrence (vivax or falciparum), time to recrudescence (new infections censored), and time to new infection (recrudescent infections censored) were assessed by the Kaplan-Meier method. Patients lost to follow-up were censored at the last time seen. When failure rates were zero, CIs were estimated with the exact binomial method with the effective sample size. All other treatments were compared against the current standard of care, which is artesunate plus mefloquine (loose tablets), and then against fixed-dose artesunate–mefloquine hydrochloride, with the log-rank test, or the Wilcoxon-Breslow test of equality if survival lines crossed. Cox regression was used to quantify risks.

Person-gametocyte weeks (PGW) were defined as the number of weeks in which gametocytaemia was patent (excluding at admission) divided by the duration of follow-up, expressed per 1000 person-weeks. The protocol was approved by the Myanmar Department of Health and by the Médecins sans Frontières ethics review board. This study is registered with clinicaltrials.gov, number NCT00902811.

### Role of the funding source

The sponsor of the study had no role in study design, data collection, data analysis, data interpretation, or writing of the report. The corresponding author had full access to all the data in the study and had final responsibility for the decision to submit for publication.

## Results

**Figure 1:**
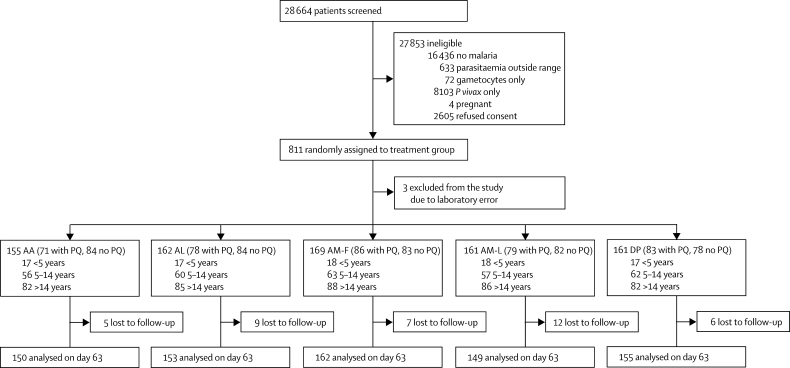
Trial profile AA=artesunate–amodiaquine. PQ=primaquine. DP=dihydroartemisinin–piperaquine. AL=artemether–lumefantrine. AM-L=loose tablet regimen of artesunate–mefloquine. AM-F=fixed-dose artesunate–mefloquine.

Figure 1 shows the trial profile. Of the patients screened for malaria, 3416 met the inclusion criteria. Most of the patients that did not agree to participate did so because they lived too far away from the study clinic. 713 of the patients who were randomly assigned to a treatment group were from Rakhine state, 66 were from Kachin state, and 32 were from Shan state. Three patients were excluded after random assignment because they were incorrectly reported as positive for *P falciparum* on day 7 and so were treated again. Of the 808 eligible patients, 397 (49·1%) also received primaquine (figure 1). All patients survived, although 39 (4·9%) did not complete the 63 day follow-up. Baseline characteristics were similar among the five treatment groups (table 1). Table 2 shows the median (IQR) and range of doses received.

**Table 1:** Baseline characteristics

		**AA**	**AL**	**AM-F**	**AM-L**	**DP**
Number of patients		155	162	169	161	161
Primaquine single dose		71	78	86	79	83
Women		55 (36%)	44 (27%)	62 (37%)	52 (32%)	50 (31%)
Age groups (years)						
	1–4	17 (11%)	17 (11%)	18 (11%)	18 (11%)	17 (11%)
	5–14	56 (36%)	60 (37%)	63 (37%)	57 (35%)	62 (39%)
	>14	82 (53%)	85 (53%)	88 (52%)	86 (53%)	82 (51%)
Mean haemoglobin g/dL (SD)		11·3 (0·26)	11·4 (0·27)	11·5 (0·25)	11·5 (0·26)	11·3 (0·24)
Anaemic (haemoglobin <10 g/dL)		48 (31%)	54 (33%)	46 (27%)	48 (30%)	50 (31%)
Geometric mean parasite count per μL (range)		7077 (521–163 881)	8709 (557–151 714)	7934 (554–177 000)	7849 (504–139 600)	7879 (546–176 000)
Mixed infections		26 (17%)	25 (15%)	21 (12%)	31 (19%)	26 (16%)
Gametocytaemia on admission		53 (34%)	54 (33%)	50 (30%)	46 (29%)	61 (38%)

AA=artesunate–amodiaquine. AL=artemether–lumefantrine. AM-F=artesunate–mefloquine fixed-dose combination. AM-L=artesunate–mefloquine loose tablets. DP=dihydroartemisinin–piperaquine.

**Table 2:** Drug doses given

	**Total target doses (mg/kg)**	**Median (IQR; range)**
Artesunate–mefloquine (loose)	12/25	12 (11·7– 12·2; 10·7–14·2); 25 (24·5–25·3; 22·3–29·5)
Artesunate–mefloquine (fixed dose)	12/26·4	14·6 (12·5– 16·7; 7·0– 30·0); 32·2 (27·5– 36·7; 15·5–66·0)
Artemether–lumefantrine	9·9/59·4	10·9 (9·8– 12·0; 7·9– 16·0); 65·4 (58·8– 72·0; 47·2–96·0)
Dihydroartemisinin–piperaquine	7·5/60	7·3 (6·5– 8·4; 4·4– 16·0); 58·2 (51·7– 67·4; 35·3–128·0)
Artesunate–amodiaquine	12/32·4	12·3 (10·7–14·3; 8·6–16·7); 33·1 (28·9–38·6; 23·2–45·0)
Primaquine	0·75	0·76 (0·7–0·82; 0·53–1·07)

**Figure 2:**
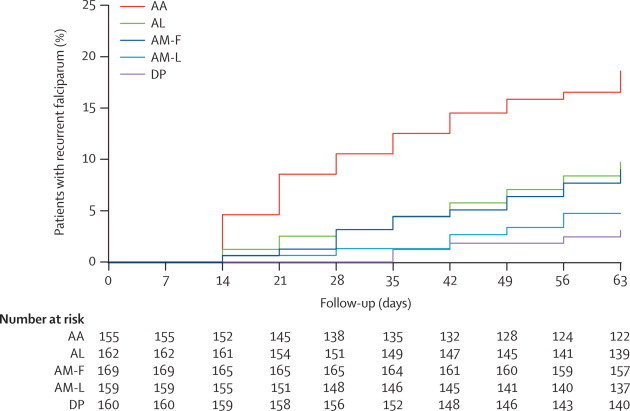
Comparative efficacy of the five ACT regimens against falciparum malaria in Myanmar Recurrences are recrudescence plus re-infections. AA=artesunate–amodiaquine. AL=artemether–lumefantrine. AM-F=fixed-dose artesunate–mefloquine. AM-L=loose tablet regimen of artesunate–mefloquine. DP=dihydroartemisinin–piperaquine.

All patients cleared parasitaemia by day 7. 69 patients had recurrence of *P falciparum* within 63 days (table 3). Recurrence was less common in patients in the fixed-dose artesunate–mefloquine group than in the fixed-dose artesunate–amodiaquine group, fixed-dose artemether–lumefantrine group, and the fixed-dose dihydroartemisinin–piperaquine group but was not significantly different to that in the loose tablet artesunate–mefloquine recipients (table 3, figure 2). Thus recurrence was less common in the two artesunate–mefloquine groups combined than in the other ACT groups (for artesunate–amodiaquine p<0.0001, for artemether–lumefantrine p=0.0097, and for dihydroartemisinin–piperaquine p=0.02). Recurrence of *P falciparum* parasitaemia was significantly (p=0·011) more common in children younger than 5 years (13 [15%] of 87 children) than in those aged 5–14 years (28 [9%] of 298), or in adults (28 [7%] of 423).

**Table 3:** Recurrence of *Plasmodium falciparum* after antimalarial treatment

	**Number by day 28; proportion (95% CI)**	**Number by day 63; proportion (95% CI)**	**Median days to event (IQR); range**	**p value for day 63 comparison with AM-L**	**p value for day 63 comparison with AM-F**
Recurrence of *P falciparum*					
AA	16; 10·5 (6·6–16·6)	28; 18·6 (13·2–25·8)	28 (17·5–42); 14–63	0·001	<0·0001
AL	5; 3·2 (1·3–7·4)	15; 9·7 (6·0–15·6)	42 (21–56); 14–63	0·09	0·01
AM-F	0; 0 (0–2·2)	5; 3·1 (1·3–7·2)	42 (35–56); 35–63	0·43	..
AM-L	2; 1·3 (0·3–5·2)	7; 4·7 (2·3–9·7)	42 (28–56); 14–56	..	0·43
DP	5; 3·2 (1·3–7·4)	14; 9·0 (5·4–14·7)	38·5 (28–56); 14–63	0·14	0·02
Recrudescent *P falciparum* infections*					
AA	12; 8·0 (4·6–13·6)	14; 9·4 (5·7–15·3)	21 (14–28); 14–49	0·002	0·0001
AL	1; 0·6 (0·1–4·5)	2; 1·4 (0·3–5·3)	42 (21–63); 21–63	0·95	0·14
AM-F	0; 0·0 (0–2·2)	0; 0 (0–2·3)	..	0·14	..
AM-L	1; 0·6 (0·1–4·5)	2; 1·3 (0·3–5·3)	31·5 (14–49); 14–49	..	0·14
DP	0; 0·0 (0–2·3)	2; 1·3 (0·3–5·2)	38·5 (35–42); 35–42	0·96	0·14
Re-infections with *P falciparum*†					
AA	2; 1·4 (0·3–5·5)	11; 8·2 (4·6–14·3)	42 (35–63); 21–63	0·02	0·05
AL	3; 1·9 (0·6–5·8)	10; 6·5 (3·6–11·8)	38·5 (28–49); 14–63	0·05	0·13
AM-F	0; 0·0 (0–2·2)	5; 3·1 (1·3–7·2)	42 (35–56); 35–63	0·58	..
AM-L	0; 0·0(0–2·5)	3; 2·1 (0·7–6·4)	56 (42–56); 42–56	..	0·58
DP	3; 1·9 (0·6–5·8)	9; 5·9 (3·1–11·1)	49 (28–56); 21–63	0·09	0·21

AM-F=artesunate–mefloquine fixed-dose combination. AM-L=artesunate–mefloquine loose tablets. AA=artesunate–amodiaquine. AL=artemether–lumefantrine. DP=dihydroartemisinin–piperaquine.

* PCR indeterminate and new infections censored.

† Recrudescent and indeterminate parasitaemias censored.

38 new infections and 20 recrudescent parasitaemias occurred by day 63. Results for nine patients were indeterminate (two artesunate–amodiaquine, three artemether–lumefantrine, three dihydroartemisinin–piperaquine, and one loose tablet artesunate–mefloquine), and data for two patients (one artesunate–amodiaquine and one loose artesunate–mefloquine) were missing. If indeterminate or missing results are treated as censored findings, then the treatment failure rate after fixed-dose artesunate–amodiaquine was significantly higher than for the other treatments (for fixed-dose artesunate–mefloquine p<0·0001, for artesunate–mefloquine loose p=0·0018, for artemether–lumefantrine p=0·0013, and for dihydroartemisinin–piperaquine p=0·0012; table 3). If indeterminate or missing results are treated as failures, then failure rates after fixed-dose artesunate–mefloquine (0%; 95% CI 0–2·3) were significantly lower than those for other treatments (for artesunate–amodiaquine [11·3%; 95% CI 7·2–17·5] p<0·0001, for artesunate–mefloquine loose [2·7%; 1·0–7·0] p=0·0355, for artemether–lumefantrine [3·4%; 1·4–7·95] p=0·0191, and for dihydroartemisinin–piperaquine [3·3%; 1·4–7·65] p=0·0207) and failure rates after fixed-dose artesunate–amodiaquine were significantly higher than with the other regimens; fixed-dose artemether–lumefantrine (p=0·0042), the loose tablet regimen of artesunate–mefloquine (p=0·0025), fixed-dose artesunate–mefloquine (p<0·0001), and fixed-dose dihydroartemisinin–piperaquine (p=0·004). If chloroquine treatment of intercurrent vivax malaria had any significant antimalarial effect it would have reduced late cases of recrudescence. Chloroquine did not affect cure rates except in the fixed-dose artesunate–amodiaquine group: there were two cases of recrudescence in the 59 (4·6%; 1·2–8·0) who did not receive chloroquine versus 12 of 96 (13·2%; 7·7–22·0) who did (p=0·04). Primaquine did not affect recrudescence rates.

The risk of new infection was more than three-times higher for patients who received artesunate–amodiaquine compared with the artesunate–mefloquine groups combined (hazard ratio [HR] 3·2; 95% CI 1·3–8·0; p=0·01) and more than twice that with artemether–lumefantrine (2·6; 1·0–6·6; p=0·04) and dihydroartemisinin–piperaquine (2·3; 0·9–6·0; p=0·08).

**Figure 3:**
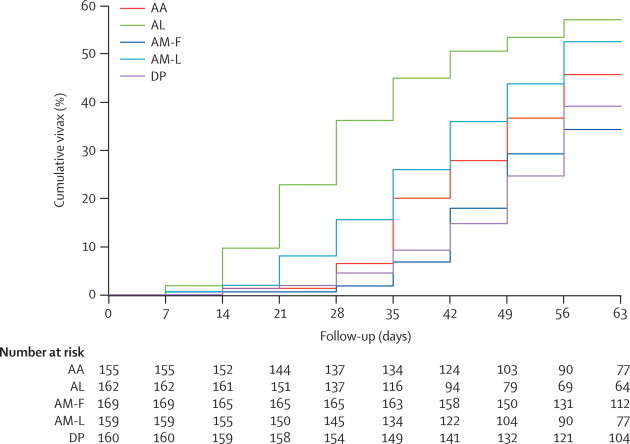
Cumulative proportion of patients with vivax malaria during follow-up AA=artesunate–amodiaquine. AL=artemether–lumefantrine. AM-F=fixed-dose artesunate–mefloquine. AM-L=loose tablet regimen of artesunate–mefloquine. DP=dihydroartemisinin–piperaquine.

129 patients (16%) had mixed infections at presentation, and these were more common in children (102 [27%] of 385) than in adults (27 [6%] of 423; risk ratio [RR] 4·2, 95% CI 2·8–6·2; p<0·0001). All patients with mixed infections responded to ACT treatment. 330 patients had *P vivax* infection during follow-up (figure 3): 259 had one episode (68 had mixed infections initially), 55 had two, and three had three. Thus the 330 patients had 404 episodes of *P vivax* during follow-up. Of the 679 patients presenting with *P falciparum* infections only, 235 (35%) had subsequent *P vivax* malaria compared with 95 (74%) of those with mixed infection initially (p<0·0001). Fewer cases of *P vivax* were recorded in patients who received fixed-dose artesunate–mefloquine than in patients who received fixed-dose artesunate–amodiaquine, fixed-dose artemether–lumefantrine, or the loose tablet regimen of artesunate plus mefloquine (table 4). The median time to the identification of *P vivax* was 35 days after fixed-dose artemether–lumefantrine, 42 days after the loose tablet regimen of artesunate plus mefloquine, 49 days after fixed-dose artesunate–amodiaquine and fixed-dose artesunate–mefloquine, and 56 days after fixed-dose dihydroartemisinin–piperaquine (p=0·0001). Thus 12 of the 14 cases of recrudescence of *P falciparum* infection after artesunate–amodiaquine happened before the median time to *P vivax* relapse for this group (table 2), which argues against a major effect of chloroquine in suppressing recrudescence. The median time to *P vivax* recurrence was shorter for children aged 0–14 years (42 days; IQR 35–56) than for adults (56; 42–63; p=0·0001). Children were nearly three times more likely to develop intercurrent vivax malaria than were adults, after adjusting for treatment and mixed infections at admission (HR 2·91; 95% CI 2·28–3·70; p<0·0001). Single-dose primaquine did not affect *P vivax* rates (data not shown).

**Table 4:** Intercurrent *Plasmodium vivax* infections

	**Number of patients**	**Number of *P vivax* infections**	**Proportion of *P vivax* infections by day 63 (95% CI)**	**p value for comparison with AM–L**	**p value for comparison with AM–F**
**Artesunate–amodiaquine**					
With *P vivax* at day 0	26	18	84 (66–96)	0·62	0·05
No *P vivax* at day 0	129	41	38 (30–48)	0·25	0·15
All	155	59	46 (38–55)	0·10	0·02
**Artemether–lumefantrine**					
With *P vivax* at day 0	25	23	..*	<0·0001	<0·0001
No *P vivax* at day 0	137	62	49 (41–58)	0·03	<0·0001
All	162	85	57 (49–65)	0·004	<0·0001
**Artesunate–mefloquine (fixed dose)**					
With *P vivax* at day 0	21	11	55 (35–77)	0·01	..
No *P vivax* at day 0	148	44	31 (24–40)	0·01	..
All	169	55	34 (28–42)	<0·001	..
**Artesunate–mefloquine (loose)**					
With *P vivax* at day 0	31	28	93 (81–99)	..	0·01
No *P vivax* at day 0	130	47	42 (33–51)	..	0·01
All	161	75	53 (45–61)	..	0·0001
**Dihydroartemisinin–piperaquine**					
With *P vivax* at day 0	26	15	68 (49–86)	0·01	0·69
No *P vivax* at day 0	135	41	34 (26–43)	0·02	0·93
All	161	56	39 (32–48)	<0·001	0·67

AM-F=artesunate–mefloquine fixed-dose combination. AM-L=artesunate–mefloquine loose tablets.

* Day 63 rate could not be calculated because all patients either lost to follow-up or relapsed by day 56.

Pooling all recurrences of falciparum or vivax malaria together provided a combined measure of efficacy and post-treatment prophylaxis, and it also addresses any possible confounding by weak activity of chloroquine against recrudescent *P falciparum*, because patients were censored at the time of any recurrence of malaria. The day 63 malaria-free rate was highest in the fixed-dose artesunate–mefloquine (64·3%; 95% CI 56·4–71·1) and the dihydroartemisinin–piperaquine (55·5%, 47·2–63·1) groups. The other three treatment groups had malaria-free rates of less than 50%, and all were significantly worse than fixed-dose artesunate–mefloquine (artesunate–amodiaquine HR 1·86, 95% CI 1·32–2·62; artemether–lumefantrine 2·46, 1·77–3·43; and the loose tablet regimen of artesunate plus mefloquine 1·79, 1·28–2·52).

**Figure 4:**
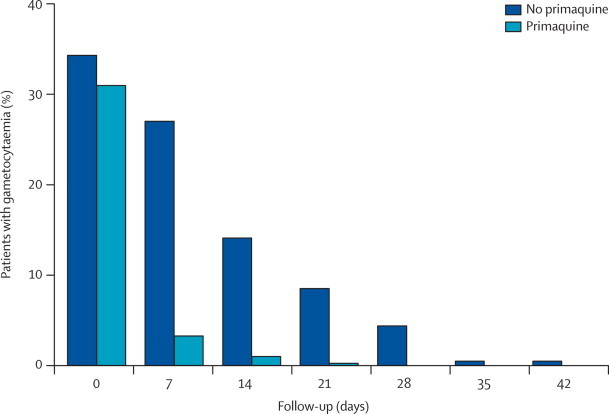
Effects of a single dose of primaquine on *Plasmodium falciparum* gametocyte carriage after artemisinin combination treatment All five ACT regimens pooled. No gametocytaemia was detected after day 42.

264 patients (33%) presented with patent gametocytaemia, which was more common in children younger than 5 years (46 [53%] of 87) than in children aged 5–14 years (121 [41%] of 298) and in adults (97 [23%] of 423; p<0·0001). Independent of age, gametocytaemia was more common among moderately anaemic patients (haemoglobin <8·0 g/dL; 49 [70%] of 70) than among patients with higher values (215 [29%] of 738; RR 2·4, 95% CI 2·0–2·9; p<0·0001). Without primaquine the treatment regimens had widely different gametocyte carriage rates (table 5). With primaquine all the treatment regimens had low gametocyte carriage, which overall was about 12-times lower than without (table 5; figure 4). New gametocytaemia on day 7 was also reduced by primaquine (one of 272 *vs* ten of 268; 0·10, 0·01–0·76; p=0.006).

**Table 5:** *Plasmodium falciparum* gametocyte carriage*

	After ACT (no primaquine)*	**p value for comparison with AM-F**	After ACT (with primaquine)*	**p value for comparison with AM-F**
Artesunate–amodiaquine	94·4	<0·001	7·50	0·43
Artemether–lumefantrine	58·2	0·009	4·65	0·86
Artesunate–mefloquine fixed-dose	29·2	..	4·02	..
Artesunate–mefloquine loose tablets	34·6	0·58	4·56	0·88
Dihydroartemisinin–piperaquine	112·8	<0·001	7·17	0·45
Total	65·51†		5·49†	

ACT=artemisinin combination therapy. combination.

* Person gametocytaemia weeks per 1000 person-weeks of follow-up.

† Comparison of all patients receiving primaquine versus patients not receiving primaquine (rate ratio 11·9; 95% CI 7·4–20·5; p≤0·0001).

246 patients (30%) had a haemoglobin concentration of less than 10·0 g/dL (mild anaemia), and 70 (9%) a concentration of less than 8·0 g/dL (moderate anaemia) at presentation. Young children were more likely to be anaemic at presentation (28 [32%] of 87) than older children (26 [9%] of 298) and adults (16 [4%] of 423; RR 5·5; 95% CI 3·6–8·4; p<0·0001). On day 63, eight (1%) of 693 patients were moderately anaemic and 72 (10%) were mildly anaemic. The mean increase of haemoglobin was similar among the five treatment groups, was unaffected by intercurrent *P vivax*, but was slightly reduced by primaquine (0·75 g/dL *vs* 1·04 g/dL; p=0·036; mean difference 0·295 g/dL; 95% CI 0·199–0·570).

599 patients (74%) reported adverse events during the study (table 6). 13 patients (1·6%) vomited within the first hour (nine patients within 30 min and four within 30–60 min), four after the loose tablet regimen of artesunate plus mefloquine, three after artesunate–amodiaquine, two after artemether–lumefantrine, two after fixed-dose artesunate–mefloquine, and two after dihydroartemisinin–piperaquine. None vomited again after treatment was repeated. Dizziness was more common after fixed dose artesunate–mefloquine than after artemether–lumefantrine (RR 1·24; 95% CI 1·03–1·48; p=0·03) and dihydroartemisinin–piperaquine (1·22; 1·02–1·45). Other side-effects were less common and were not related to a specific treatment. The only side-effect attributable to primaquine was abdominal pain (table 7). There were no cases of urticaria or other potentially serious skin reactions, convulsions, behaviour disturbances, blackwater fever, or severe anaemia (<50 g/L).

**Table 6:** Number of patients reporting side-effects at least once during study

	**AA (n=155)**	**AL (n=162)**	**AM-F (n=169)**	**AM-L (n=161)**	**DP (n=161)**	**Total (n=808)**	p value*
Dizziness	91 (59%)	86 (53%)	111 (66%)	104 (65%)	87 (54%)	479 (59%)	0·06
Nausea	27 (17%)	28 (17%)	33 (20%)	30 (19%)	28 (17%)	146 (18%)	0·98
Anorexia	29 (19%)	17 (11%)	25 (15%)	21 (13%)	22 (14%)	114 (14%)	0·34
Diarrhoea	17 (11%)	12 (7%)	11 (7%)	16 (10%)	20 (12%)	76 (9%)	0·32
Abdominal pain	20 (13%)	23 (14%)	23 (14%)	26 (16%)	19 (12%)	111 (14%)	0·83
Palpitations	35 (23%)	25 (15%)	38 (23%)	41 (26%)	32 (20%)	171 (21%)	0·23
Sleeplessness	22 (14%)	14 (9%)	25 (15%)	24 (15%)	18 (11%)	103 (13%)	0·33
Headache	2 (1%)	2 (1%)	0	0	2 (1%)	6 (1%)	0·38†
Vomiting in first 24 h	7 (5%)	6 (4%)	9 (5%)	16 (10%)	10 (6%)	48 (6%)	0·18

AA=artesunate–amodiaquine. AL=artemether–lumefantrine. AM-F=artesunate–mefloquine fixed-dose combination. AM-L=artesunate–mefloquine loose tablets. DP=dihydroartemisinin–piperaquine.

* For comparison of frequency across the five treatment groups, adjusted for primaquine allocation.

† p value for differences between treatment groups not adjusted for primaquine allocation.

**Table 7:** Number of patients reporting side-effects after treatment with primaquine

	**With primaquine (n=397)**	**No primaquine (n=411)**	**Total (n=808)**	p value*
Dizziness	239 (60%)	240 (58%)	479 (59%)	0·61
Nausea	78 (20%)	68 (17%)	146 (18%)	0·26
Anorexia	48 (12%)	66 (16%)	114 (14%)	0·11
Diarrhoea	31 (8%)	45 (11%)	76 (9%)	0·13
Abdominal pain	64 (16%)	47 (11%)	111 (14%)	0·05
Palpitations	86 (22%)	85 (21%)	171 (21%)	0·73
Sleeplessness	50 (13%)	53 (13%)	103 (13%)	0·90
Headache	4 (1%)	2 (1%)	6 (1%)	0·44†
Vomiting in first 24 h	25 (6%)	23 (6%)	48 (6%)	0·69

* For comparison between primaquine allocation, adjusted for treatment groups.

† p value not adjusted for differences between treatment groups.

## Discussion

Control and ultimately elimination of falciparum malaria depends on providing effective and well tolerated medicines and minimising transmission. In Myanmar, our large study shows that artesunate–mefloquine, the first-line treatment in the main study region since 1996, remains very effective. The new fixed-dose artesunate–amodiaquine was well tolerated, but was not effective enough to be recommended as a first-line treatment. The other available ACTs were all well tolerated and highly effective, suggesting good adherence to the prescribed treatments, although only the first dose was observed (ie, the normal context of use). Addition of a single dose of primaquine to ACT regimens has a very large additional effect on gametocytaemia, and therefore on malaria transmission potential, making any residual differences in gametocyte carriage after the primary infection between the five ACT regimens insignificant.

From an operational perspective, fixed-dose combinations are simple to give and avoid monotherapy, thereby protecting against resistance. Findings from our previous studies have shown that artesunate–mefloquine, the first-line treatment in Myanmar since 1996, is very effective.^2^, ^3^, ^4^, ^10^ The new fixed-dose artesunate–mefloquine has the greatest efficacy of all the ACTs assessed.^5^ Artesunate–mefloquine regimens were also associated with greater post-treatment prophylaxis than the other drugs,^12^ although differences with dihydroartemisinin–piperaquine were not significant. Although artesunate–mefloquine regimens were associated with slightly more dizziness than were fixed-dose artemether–lumefantrine or dihydroartemisinin–piperaquine,^2^, ^3^, ^4^, ^9^, ^10^, ^13^, ^14^ high cure rates argue against a significant effect on adherence.

Malaria transmission in Myanmar is seasonal and generally low, but access to effective drugs is limited, and the clinical epidemiology is similar to that of higher-transmission settings with a high prevalence of anaemia, high rates of *P falciparum* gametocytaemia, and substantial discrepancy between cure rates in adults and children.^2^, ^3^, ^4^, ^10^ The artemisinin component of ACTs reduces gametocyte carriage,^6^, ^7^, ^15^ and patients who received artesunate–mefloquine regimens had the lowest rates of gametocyte carriage after treatment, probably because of the higher dose of the artemisinin derivative compared with artemether–lumefantrine and dihydroartemisinin–piperaquine, and the greater antimalarial effect of mefloquine compared with amodiaquine. The use of ACTs in parts of Rakhine state since 1996^2^, ^3^, ^4^, ^10^ has resulted in a gradual but substantial decrease in falciparum malaria in regions where the drugs are available. The high gametocyte carriage rates probably contribute to differences in the effect of ACTs on malaria transmission and thus incidence in Myanmar compared with that in other areas in the region, such as the western border of Thailand, where gametocyte carriage rates are low, treatment delays are brief, and, as a result of ACT deployment, falciparum malaria incidence has fallen substantially.^16^ Greater coverage of effective drug treatments with more potent transmission-blocking activity is expected to result in greater effects on malaria incidence.

The 8-aminoquinolines have a rapid and powerful sterilising effect on mature gametocytes.^17^, ^18^ Single-dose primaquine has been widely recommended in the past, and used extensively in eradication campaigns; however, its safety profile has not been well characterised.^19^ Primaquine causes abdominal discomfort, particularly if taken on an empty stomach, and potentially serious haemolytic anaemia in patients with glucose-6-phosphate dehydrogenase (G6PD) deficiency.^20^ Single doses have been judged safe, particularly when prevalent genotypes confer mild G6PD deficiency, although a recent study has challenged this assumption.^21^ In our study there were no severe episodes of haemolysis, although we did note a small but significant adverse effect on recovery from anaemia. We did not test for G6PD deficiency, and more frequent measurements of haemoglobin concentrations were not possible.

Mixed *P falciparum* and *P vivax* infections were common. 330 patients (41%) had at least one episode of vivax malaria in the 63 day follow-up. The risk was particularly high in young children—56 children (64%) younger than 5 years had vivax malaria after acute falciparum malaria. This compares with *P falciparum* recrudescence rates of less than 10%. Treatment of vivax relapses with chloroquine might have provided weak additional activity against *P falciparum*, suppressing some late recrudescence, particularly for the less effective artesunate–amodiaquine. Although because most recrudescence happened within 4 weeks, and most vivax infections were not noted until after 5 weeks, this effect is unlikely to have been large. Thus for the four highly effective ACT regimens the main clinical problem after effective treatment of acute falciparum malaria in Myanmar is recurrent *P vivax* malaria not *P falciparum*.^22^ Recurrence of vivax malaria was over four times more likely than was recurrence of falciparum malaria, emphasising the public health importance of vivax infections after falciparum malaria. If safe radical treatment was available, then these very high rates would argue for radical treatment of all malaria in this region where mixed infections are so common.

This is, to our knowledge, the first comparison of all currently available fixed-dose artemisinin combination treatments for falciparum malaria. Our findings suggest that artesunate–mefloquine, artemether–lumefantrine, or dihydroartemisinin–piperaquine are all good treatments of falciparum malaria in Myanmar. The new fixed dose artesunate–mefloquine had the highest cure rates, the lowest rates of gametocyte carriage, and the most effective suppression of *P vivax* malaria. Adverse effects were generally mild, despite the high doses received by some patients, and did not substantially affect adherence. Although giving the entire 25 mg/kg dose of mefloquine as loose tablets was directly observed, suppression of recurrent vivax malaria was significantly worse than with the fixed combination, suggesting better absorption of mefloquine with the latter.^5^, ^23^, ^24^ The addition of a single gametocytocidal dose of primaquine was well tolerated and highly effective and did not cause serious adverse effects, although more studies to characterise the safety with different G6PD deficiency genotypes are needed. The addition of a single dose of primaquine to ACTs could have a major effect on malaria transmission from treated patients, and could have a crucial role in elimination programmes.^25^
